# Selection of the optimal chelator for labeling of DARPin Ec1 with gallium-68 for PET imaging of EpCAM expression

**DOI:** 10.1186/s41181-025-00347-6

**Published:** 2025-05-30

**Authors:** Anzhelika Vorobyeva, Moeen-ud Din, Alexey Schulga, Elena Konovalova, Ayman Abouzayed, Olga Bragina, Ruonan Li, Torbjörn Gräslund, Sergey M. Deyev, Maryam Oroujeni

**Affiliations:** 1 Department of Immunology, Genetics and Pathology, Uppsala University, 751 85 Uppsala, Sweden; 2 Molecular Immunology Laboratory, Shemyakin and Ovchinnikov Institute of Bioorganic Chemistry, Russian Academy of Sciences, 117997 Moscow, Russia; 3 Department of Medicinal Chemistry, Uppsala University, 75183 Uppsala, Sweden; 4 Department of Nuclear Medicine, Cancer Research Institute, Tomsk National Research Medical Center Russian Academy of Sciences, 634050 Tomsk, Russia; 5 Research Centrum for Oncotheranostics, Research School of Chemistry and Applied Biomedical Sciences, Tomsk Polytechnic University, 634050 Tomsk, Russia; 6 Department of Protein Science, KTH Royal Institute of Technology, 10691 Stockholm, Sweden

**Keywords:** Epithelial cell adhesion molecule (EpCAM), Designed ankyrin repeat proteins (DARPin), ^68^Ga, SKOV-3 xenograft, PET imaging

## Abstract

**Background:**

Epithelial cell adhesion molecule (EpCAM) is a transmembrane glycoprotein, which is overexpressed in several types of malignancies. Designed ankyrin repeat protein (DARPin) Ec1 is a 19 kDa engineered scaffold protein that binds with high affinity to EpCAM. Radiolabelled Ec1 might be used as a companion diagnostic for the selection of patients for personalized therapy. This study aimed to investigate the influence of different radiometal-chelator complexes on the biodistribution and imaging contrast of ^68^Ga-labelled Ec1. To investigate this, two macrocyclic chelators, 1,4,7-triazacyclononane-N,N,N-triacetic acid (NOTA) and 1-(1,3-carboxypropyl)-1,4,7-triazacyclononane-4,7-diacetic acid (NODAGA) were conjugated to the C-terminus of the Ec1. The previously developed DARPin Ec1 conjugated to 1,4,7,10-tetraazacylododecane-1,4,7,10-tetraacetic acid (DOTA) was used as a comparator.

**Results:**

All Ec1 variants were successfully labelled with ^68^Ga. The use of NOTA and NODAGA provided twice higher radiochemical yield and improved label stability compared to DOTA. All labelled Ec1 variants bound to the EpCAM-expressing cells with nanomolar affinity and preserved targeting specificity in vitro and in vivo. Biodistribution studies in mice bearing EpCAM-expressing SKOV-3 xenografts showed that [^68^Ga]Ga-Ec1-NOTA had lower uptake in most normal organs while maintaining tumor uptake. Among all variants, [^68^Ga]Ga-Ec1-NOTA showed the lowest liver uptake, with no significant differences in tumor uptake. Additionally, [^68^Ga]Ga-Ec1-NOTA provided the highest tumor-to-blood ratio compared to [^68^Ga]Ga-Ec1-DOTA and [^68^Ga]Ga-Ec1-NODAGA.

**Conclusion:**

[^68^Ga]Ga-Ec1-NOTA is the preferred radioconjugate for PET imaging of EpCAM expression.

## Introduction

One of the promising druggable cancer-associated molecular targets is epithelial cell adhesion molecule (EpCAM, also known as CD326). EpCAM is a transmembrane glycoprotein consisting of 291 amino acids, including a large glycosylated and disulfide-bonded extracellular domain, a single transmembrane helix, and a short cytoplasmic domain, which is involved in intracellular signaling, regulating migration, proliferation, and differentiation (Liu et al. 2022a).

The high-level expression of EpCAM in various carcinomas (e.g. prostate, ovarian, lung, colon, and breast cancers) and metastases, along with its correlation to clinical outcomes, has established it as both a prognostic marker and a therapeutic target (Went et al. 2006). However, EpCAM’s prognostic value varies depending on the tumor type. EpCAM plays a critical role in tumorigenesis and metastasis of carcinomas and can be a potential prognostic marker for immunotherapeutic strategies (Gires et al. 2020). Molecular imaging of cell-surface proteins might facilitate the diagnosis and stratification of patients for targeted treatment. Radionuclide molecular imaging of molecular targets is a non-invasive tool assisting physicians to stratify patients for the specific targeted therapy. Clinical examples include imaging of human epidermal growth factor receptor 2 (HER2) using radiolabeled affibody molecules to select patients for HER2-targeted therapy (Altena et al. 2023; Eissler et al. 2024; Altena et al. 2024), prostate-specific membrane antigen (PSMA) imaging to select patients for PSMA-targeted radioligand therapy (Hofman et al. 2020) or imaging of B7H3 for B7H3-targeted therapies (Xia et al. 2025). This technique can solve the major drawbacks associated with the use of biopsy-based methods such as invasiveness and target expression heterogeneity. The clinical relevance of EpCAM imaging is emphasized by its heterogeneous expression across various cancer types and within individual tumors. This heterogeneity presents challenges in accurately identifying suitable candidates for EpCAM-targeted therapies and requires careful evaluation to guide clinical decision-making. Traditional pathological assessment by biopsy may not fully capture the heterogeneity of EpCAM expression. Additionally, the EpCAM expression can be dynamic in primary and metastatic tumors (Cui et al. 2022). This may potentially lead to misclassification of tumors and suboptimal treatment strategies. Molecular imaging offers a non-invasive means to assess the spatial and temporal distribution of EpCAM expression in vivo. Incorporating molecular imaging into clinical workflows might enhance the precision of EpCAM-targeted therapies, potentially improving patient outcomes. 

Previously developed probes for radionuclide molecular imaging of EpCAM are mainly based on monoclonal antibodies (mAbs) (Kosterink et al. 1995; Breitz et al. 1997; Hall et al. 2012; Warnders et al. 2016). Although the use of the radiolabeled antibodies is straightforward, it is associated with a risk of false-positive results due to nontarget-mediated retention in tumors in part because of the enhanced permeability and retention (EPR) effect (Maeda et al. 2000; McLarty et al. 2009; Lub-de Hooge et al. 2004; Wijngaarden et al. 2024).. Moreover, the low tumor extravasation rate and long serum half-life of full-length antibodies result in high background and low imaging contrast of radionuclide imaging even several days after administration (Wu 2014).

The use of small targeting proteins have earlier been found suitable as an alternative to overcome the limitations associated with the use of mAbs. The reduction in the size of the targeting agent facilitates extravasation, enabling efficient and quick accumulation in tumors (Eder et al. 2010; Liu et al. 2022c; Xu et al. 2025). Moreover, the non-bound targeting agents are rapidly excreted from blood circulation, resulting in a high imaging contrast even on the day of administration (Garousi et al. 2020). Examples of small targeting agents are the engineered scaffold proteins (ESPs), for example affibody molecules, albumin-binding domain-derived affinity proteins (ADAPTs), and designed ankyrin repeat proteins (DARPins). They have demonstrated the capacity to provide high-contrast in vivo imaging of different molecular targets at the day of injection in preclinical and clinical studies (Krasniqi et al. 2018; Alhuseinalhudhur et al. 2023; Luo et al. 2022; Bragina et al. 2021; Bragina et al. 2022; Houvast et al. 2024).

DARPins comprise a class of small proteins based on tightly packed repeats of 33-amino acids, forming a β-turn and two antiparallel α-helices. They are characterized by high robustness, high thermal and chemical stability, high water solubility and efficient production in prokaryotic hosts with low manufacturing costs (Binz et al. 2003, 2004; Steiner et al. 2008; Interlandi et al. 2008). DARPins binding to tumor-associated molecular targets such as human epidermal growth factor receptor 2 (HER2), epithelial cell adhesion molecule (EpCAM), epidermal growth factor receptor (EGFR), vascular endothelial growth factor (VEGF) and hepatocyte growth factor (HGF) have been described (Binz et al. 2017; Stefan et al. 2011; Zahnd et al. 2006; Steiner et al. 2008).

DARPins are currently the only class of ESPs that has been used to successfully generate binders to EpCAM. Clinical trials have demonstrated that DARPin-based radionuclide imaging probes can successfully target and visualize cancer-associated upregulation of proteins in malignancies, for example DARPin G3 targeting HER2, and Ec1 targeting EpCAM (Bragina et al. 2022; Zelchan et al. 2024). The DARPin Ec1 consists of five ankyrin repeats and has a molecular weight of 18.6 kDa. It has a strong affinity to EpCAM (K_D_ = 68 pM) (Stefan et al. 2011). Radiolabeling of Ec1 with different nuclides, such as technetium-99 m, iodine-125, or indium-111, has enabled the development of imaging probes demonstrating specific visualization of EpCAM expression in preclinical models of pancreatic, ovarian, prostate and triple-negative breast cancers (Deyev et al. 2020; Vorobyeva et al. 2020a, b; Deyev et al. 2021). A Phase I clinical study demonstrated that injections of Ec1, radiolabelled with technetium-99 m tricarbonyl, is safe and well tolerated, and that the tracer can visualize EpCAM expression in patients with lung cancer (Zelchan et al. 2024).

Visualization of EpCAM expression using molecular imaging techniques, such as single photon emission computed tomography (SPECT) and positron-emission tomography (PET), can overcome many limitations associated with the use of biopsy-based methods. PET is commonly used in oncology for cancer staging, treatment planning, and monitoring response to therapy. One of the significant advantages of PET is its ability to provide quantitative information about physiological processes, allowing for the measurement of metabolic rates, and receptor densities. Importantly, PET can provide superior sensitivity and quantitation accuracy compared to SPECT (Rahmim and Zaidi 2008). Among the possible positron emitting radioisotopes such as ^55^Co (T_1/2_ = 17.5 h), ^64^Cu (T_1/2_ = 12.7 h) and ^89^Zr (T_1/2_ = 78.4 h), which can be used to provide PET images on the day after injection, gallium-68 (^68^Ga, T_1/2_ = 68 min, β^+^ abundance 90%, E_β+max_ = 1880 keV) is a suitable positron-emitting radionuclide for clinical PET imaging of different cancerous targets on the day of injection. The major advantage of this radionuclide compared to many others lies in its accessibility from a ^68^Ge/^68^Ga generator system, which provides a non-cyclotron-based and cost-effective source of the isotope. This offers hospitals convenient on-site access to a GMP-compliant diagnostic PET radionuclide, eliminating the need for local cyclotron facilities. The short half-life of this radionuclide fits well with rapidly cleared imaging agents such as ESPs, resulting in a low absorbed dose burden to the patients. Considering these advantages, there has been significant interest in ^68^Ga-containing radiopharmaceuticals (Fani et al. 2008; Velikyan 2014). ^68^Ga-based radiopharmaceuticals consist of gallium-68 bound to a chelator, which is linked to a molecule designed to target cell-surface receptors on tumors. The optimal chelator for radiopharmaceutical applications should form thermodynamically stable and/or kinetically inert complex to prevent any premature ligand-exchange reactions or hydrolysis in the body. This is particularly important for Ga (III), which can bind to transferrin in blood at two transferrin binding sites with high thermodynamic stability (log *K*_*1*_ = 21.43 and log *K*_*2*_ = 20.57) (Harris and Pecoraro 1983). Therefore, gallium-chelator complexes should be sufficiently inert to transchelation in vivo. Macrocyclic chelators such as NOTA, NODAGA, and DOTA are commonly used for labeling of peptides and proteins with gallium-68 (Kostelnik and Orvig 2019). Even though DOTA is a suboptimal chelator for gallium, as it has a large cavity and the thermodynamic stability of the DOTA-gallium complex (log *K*_DOTA-Ga(III)_ = 21.3) is lower than the stability of the NOTA-gallium complex (log *K*_NOTA-Ga(III)_ = 31.0) (Wadas et al. 2010), DOTA is commonly used for gallium-66/67/68 labeling (Kostelnik and Orvig 2019) and is a convenient choice for theranostic applications with other radiometals. NOTA and NODAGA chelators, containing three nitrogen donor atoms, are smaller than DOTA, containing four nitrogen donor atoms. NODAGA is a derivative of NOTA containing one additional carboxy group. After one of the carboxylic acid groups is used for conjugation to a targeting molecule, two, three, and three groups remain to saturate the hexadentate coordination of gallium for the NOTA, NODAGA, and DOTA chelators, respectively. Thus, the coordination geometry of their complexes with gallium-68 is different. The characteristics of the radiometal-chelator complex, such as complex geometry, lipophilicity, and overall charge, can influence the biodistribution of targeted radiopharmaceuticals. Several studies have demonstrated how minor changes in the chelator structure, conjugation linker, and the site of its conjugation to the peptide or protein, can influence the uptake in normal organs and tumor-targeting properties of imaging agents labeled with radiometals (Asti et al. 2014; Strand et al. 2013; Rinne et al. 2019a, Mitran et al. 2019). This means that the biodistribution and tumor-targeting properties of the targeting agents could be improved by the alteration of their structural features. 

Thus, the main aim of this study was to evaluate how the modification of radiometal-chelate complex would influence the biodistribution and tumor-targeting properties of Ec1 labelled with gallium-68 for visualizing the EpCAM expression. To construct imaging agents labelled with generator-produced gallium-68, the maleimide derivatives of the macrocyclic chelators, DOTA, NOTA and NODAGA, were site-specifically coupled to the C-terminal cysteine in Ec1. The resultant Ec1 constructs were labelled with gallium-68. In vitro experiments to test binding specificity, affinity, and internalization were performed using EpCAM-expressing cell lines. Biodistribution of ^68^Ga-labelled Ec1 variants was studied in mice bearing EpCAM-expressing SKOV-3 xenografts. The uptake in EpCAM-negative Ramos xenografts was used to test the specificity of ^68^Ga-labelled Ec1 variants in vivo.

## Materials and methods

### General

Gallium‐68 was obtained by fractionated elution of the ^68^Ge/^68^Ga generator (Eckert and Ziegler AG, Berlin, Gemany) with 0.1 M HCl. The eluate with the highest radioactivity concentration was used for labeling. The iTLC analysis of radiochemical yield and purity was performed using CR35 BIO Plus Storage Phosphor System and AIDA image analysis software (from ElysiaRaytest, Bietigheim-Bissingen, Germany). The activity from cell and animal samples was measured using an automated gamma-spectrometer equipped with a 3-inch NaI (TI) well detector (2480 Wizard, Wallac, Turku, Finland). Radioactivity during labeling was measured using a dose calibrator RDC-VIK-202 (COMECER Netherlands, 8501-XC, Jourse, Netherlands) equipped with an ionizing chamber.

In vitro cell studies were performed using the ovarian cancer cell lines, SKOV-3 and OVCAR-3, which were purchased from the American Type Culture Collection (ATCC). Ramos lymphoma cells (ATCC) were used to establish EpCAM-negative xenografts. SKOV-3 and Ramos cells were cultured in RPMI medium (Biochrom, Berlin, Germany) containing 10% fetal bovine serum (FBS) (Merck, Darmstadt, Germany), 2 mM L-glutamine, 100 IU/mL penicillin, and 100 µg/mL streptomycin (all from Biochrom, Berlin, Germany). OVCAR-3 cells were cultured in RPMI medium containing 20% FBS, 2 mM L-glutamine, 100 IU/mL penicillin, 100 g/mL streptomycin, and 0.01 mg/mL bovine insulin (Sigma-Aldrich, St. Louis, MO, USA).

Statistical analysis was performed using Prism 10.4.1 software (GraphPad Software Inc). A 2-tailed unpaired t-test was applied to find a significant difference for comparison of two sets of data. To determine significant differences (p < 0.05) of more than two sets of data, a one-way ANOVA with Tukey’s multiple comparisons was used.

### Production, purification, and characterization of Ec1 conjugated to DOTA, NOTA or NODAGA chelators

Ec1 having a histidine-glutamate (HE)_3_ tag at the N-terminus and three glutamates followed by a cysteine at the C-terminus ((HE)_3_-Ec1-E_3_C) was produced and conjugated to the chelators as described previously (Deyev et al. 2021). For conjugation of different chelators (malemide derivatives of DOTA, NOTA and NODAGA) (molecular weights 787, 685 and 757 mg/mmol, respectively) to Ec1, (HE)_3_-Ec1-E_3_C (1.5 mg, 80 nmol, 400 μL in PBS, pH 8) was incubated with a 100-fold molar excess of dithiothreitol (DTT) (1.28 mg, 8.2 μmol, 26 μL in PBS, pH 8.0), final DTT concentration 19 mM, for 60 min at 40 ℃. To remove DTT, the reaction mixture was purified using a NAP-5 column, pre-equilibrated with degassed 0.2 M ammonium acetate (NH_4_OAc), pH 6.5. Next, the fractions containing the protein (0.45 mg, 24 nmol, 480 μL) were incubated with a tenfold molar excess of maleimide-DOTA (190 μg, 240 nmol, 38 μL of 5 mg/mL in 0.2 M NH_4_OAc, pH 6.5), maleimide-NOTA (164 μg, 240 nmol, 32 μL of 5 mg/mL in 0.2 M NH_4_OAc, pH 6.5) and maleimide-NODAGA (182 μg, 240 nmol, 36 μL of 5 mg/mL in 0.2 M NH_4_OAc, pH 6.5) at 40 ℃ for 2 h. To remove the unconjugated chelator, the reaction mixture was purified using a NAP-5 column with 0.2 M NH_4_OAc, pH 6.5. The protein concentration was measured using a DS-11 spectrophotometer (DeNovix, Wilmington, DE, USA). The Ec1 variants conjugated to chelators (termed Ec1-DOTA, Ec1-NOTA and Ec1-NODAGA, respectively) were stored in 0.2 M NH_4_OAc (pH 6.5) at −20 ℃ before labeling with radiometal. The molecular weight of the conjugates was measured by liquid chromatography–electrospray ionization–mass spectrometry (LC–ESI–MS) on a 6520 Accurate Q-TOF LC/MS (Agilent, USA).

### Radiolabeling with ^68^Ga and in vitro stability

For labeling with ^68^Ga, 30 µg of Ec1 conjugate was mixed with 1.25 M of NaOAc, pH 3.6 (100–150 µL). A generator eluate (100–150 µL, 60 MBq) was added. The reaction mixture was thoroughly vortexed and incubated for 25 min at 60 ℃. The radiochemical yield was evaluated using iTLC developed with 0.2 M citrate buffer pH 2.0. To remove unbound activity, 1000-fold molar excess of EDTANa_4_ (10 μL of 38 mg/mL) was added to the reaction mixture. Purification was performed using a NAP-5 column, pre-equilibrated and eluted in PBS.

The in vitro stability test of Ec1 variants labeled with ^68^Ga was performed by incubating purified radioconjugates with a 1000-fold molar excess of EDTA (10 mg/mL) at 37 ℃. The control samples were incubated in PBS. The test was run in triplicates.

The stability in serum was analyzed by mixing the purified radioconjugates (3 μg in 47 μL) or ^68^Ga[Ga]Cl_3_ with murine serum or PBS (47 μL) as a control. The samples were incubated for 3 h at 37 °C and analyzed by iTLC at 1 h and 3 h. After 3 h, the samples were loaded on NAP-5 column pre-equilibrated with 1% BSA in PBS and eluted with PBS. The following fractions were collected: (1) fraction containing high-molecular weight components eluted in 900 μL, corresponding to the protein-associated activity in Table 3; and (2) fraction containing low-molecular weight components eluted in 1800 μL. The activity in fractions and in the NAP-5 columns were measured using an automated gamma-spectrometer. The sum of two fractions and the residual activity in NAP-5 column were taken as 100%, and the protein-associated activity was calculated. The experiment was performed in duplicate.

To cross-validate radio-iTLC data, reverse phase-HPLC was performed conducted on an Elite LaChrom system (Hitachi, VWR, Darmstadt, Germany) consisting of an L-2130 pump, a UV detector (L-2400), and a radiation flow detector (Bioscan, Washington, DC, USA) coupled in series was used. Analysis of the purity of radiolabelled compound was performed using an analytical column (Vydac RP C18 column, 300 Å; 3 × 150 mm; 5 µm). HPLC conditions were as follows: A = 10 mM TFA/H_2_O, B = 10 mM TFA/acetonitrile, UV-detection at 220 nm, gradient elution: 0–15 min at 5% to 70% B, 15–18 min at 70 to 95% B, 19–20 min at 5% B, and a flow rate was 1.0 mL/min.

### In vitro studies

The binding specificity of Ec1 conjugates labelled with gallium-68 to EpCAM-expressing SKOV-3 and OVCAR-3 cells was evaluated as described previously (Vorobyeva et al. 2020a). A 100-fold molar excess of non-labelled Ec1 (200 nM in 500 µL) was added to the blocked group of cells to saturate EpCAM receptors, then the radiolabelled DARPin radioconjugates (4 nM in 500 µL) were added to both groups (2 nM final concentration).

For half-maximal inhibitory assay (IC_50_), 5 × 10^5^ SKOV-3 cells/well were seeded in a 12-well plate. On the next day, the medium was removed, 250 μL of fresh medium was added to every well, followed by the addition of 250 μL of the [^nat^Ga]Ga-Ec1-X (X = NOTA, DOTA and NODAGA) and [^111^In]In-Ec1-DOTA (500 μL). The final concentration of the [^nat^Ga]Ga-Ec1-X conjugates ranged between 0 and 400 nM and the final concentration of [^111^In]In-Ec1-DOTA was 2 nM. The cells were incubated at 4 °C for 4 h. The supernatant was collected and the cells were washed with cold PBS, which was collected to the same tube. The cells were collected using a trypsin–EDTA solution. The activity in the samples was measured. The data was analysed using the nonlinear regression model in GraphPad Prism 10.4.1. Statistical analysis was performed using one-way ANOVA test with Bonferroni’s correction. The experiment was performed in parallel using the cells from the same passage and the same solution of [^111^In]In-Ec1-DOTA.

Cellular processing of Ec1 radioconjugates on SKOV-3 and OVCAR-3 cells during continuous incubation was studied by an acid-wash method (Wållberg and Orlova 2008). Three dishes per time point were used. Radioconjugate (2 nM in 1 mL) was added to the dishes and cells were incubated at 37 °C in a humidified incubator for 0.5, 1, 2 and 3 h. 

### In vivo studies

Animal experiments were performed according to the national legislation for work with laboratory animals and the permit granted by the Ethical Committee for Animal Research in Uppsala (permit 5.8.18–00473/2021, approved 26 February 2021). Four mice per data point were used in biodistribution experiments.

Biodistribution of the ^68^Ga-labelled Ec1 conjugates was studied in female Balb/c nu/nu mice bearing EpCAM-positive SKOV-3 xenografts. To establish xenografts, SKOV-3 cells (10^7^ cells/mouse) were subcutaneously injected on the right hind leg of mice. For in vivo specificity, Ramos cells (6 × 10^6^ cells/mouse) were subcutaneously implanted on the left hind leg of mice. The experiments were performed two to three weeks after cell implantation. The average animal weight was 19.1 ± 0.7 g. The average tumor weight was 1.04 ± 0.41 g and 0.63 ± 0.52 g for SKOV-3 and Ramos xenografts, respectively. The biodistribution was measured 3 h after injection. Mice were injected with the ^68^Ga-labelled Ec1 variants ([^68^Ga]Ga-Ec1-DOTA (9.3 ± 0.3 µg, 660 ± 21 KBq); [^68^Ga]Ga-Ec1-NOTA (9.3 ± 0.5 µg, 680 ± 37 KBq); [^68^Ga]Ga-Ec1-NODAGA (9.4 ± 0.2 µg, 719 ± 17 KBq) in 100 µL of PBS) into the tail vein. To test EpCAM-mediated accumulation, a group of animals bearing Ramos xenografts was injected with the same peptide dose and activity and the biodistribution was measured 3 h after injection. Mice were euthanized by intraperitoneal injection of anaesthetic solution following by a heart puncture, and collection of blood. Organs and tissues were collected and weighed. The organ radioactivity was measured using a gamma spectrometer. Uptake values for organs were calculated as the percentage of injected dose per gram of tissue (%ID/g).

To confirm the biodistribution results, a small animal PET/CT imaging was performed. Two mice bearing SKOV-3 xenografts were intravenously injected with [^68^Ga]Ga-Ec1-DOTA (9.6 µg, 4.51 MBq) or [^68^Ga]Ga-Ec1-NOTA (9.6 µg, 4.50 MBq) (n = 2, 9.6 ± 0.0 µg, 4.51 ± 0.01 MBq) and imaged three hours after injection. CT acquisition was performed using nanoScan PET/CT (Mediso Medical Imaging Systems Ltd., Hungary) immediately after PET acquisition using the same bed position. The PET scans were performed for 60 min, followed by CT examination at the following parameters: CT-energy peak of 50 keV, 670 A, 480 projections, 2.29 min acquisition time. The PET data were reconstructed into a static image using Tera-Tomo™ 3D reconstruction engine. CT raw files were reconstructed in real time using Filter Back Projection in Nucline 2.03 Software (Mediso Medical Imaging Systems, Hungary). PET and CT files were fused and analyzed using Nucline 2.03 Software (Mediso Medical Imaging Systems, Hungary) and were presented as maximum intensity projections (MIP) in RGB colour scale.

## Results

### Production, purification, and characterization of Ec1 conjugated to DOTA, NOTA or NODAGA chelators

**Fig. 1:**
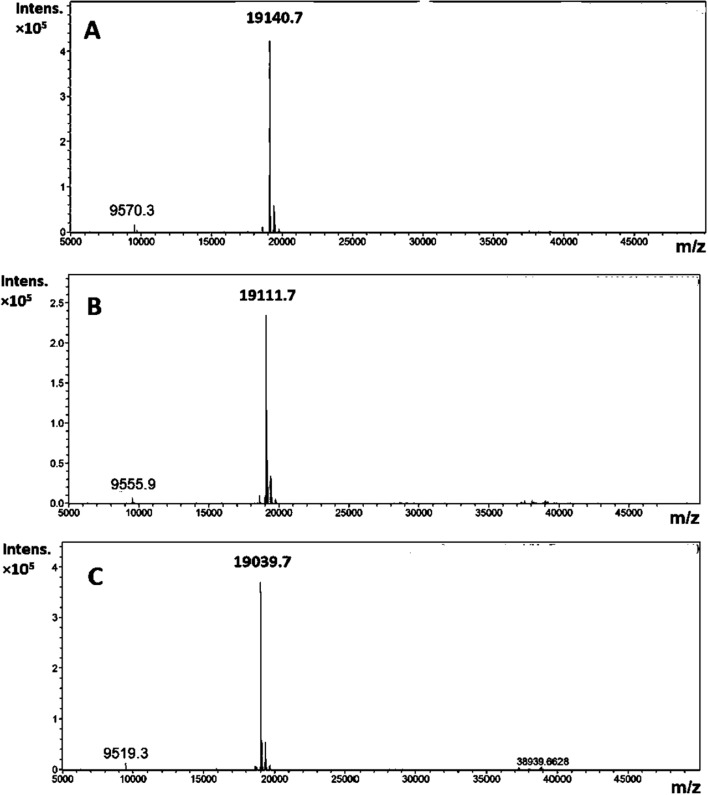
ESI–MS spectra of **A** Ec1-DOTA, **B** Ec1-NODAGA and **C** Ec1-NOTA Calculated molecular weight was 19,141, 19,111 and 19,039 for Ec1-DOTA, Ec1-NODAGA and Ec1-NOTA, respectively. Found molecular weight was 19,140.7, 19,111.7 and 19,039.7 for Ec1-DOTA, Ec1-NODAGA and Ec1-NOTA, respectively

The Ec1 was recombinantly expressed and purified followed by coupling of the chelators DOTA, NOTA, and NODAGA. According to mass spectrometry analysis (Fig. 1), the coupling of maleimide-chelators provided successful conjugation to Ec1, with the obtained molecular weights after conjugation matching the theoretical values within 1 Da (Fig. 1).

### Radiolabeling of Ec1 variants with gallium-68 and in vitro stability

All variants were successfully labeled with gallium-68 (Table 1). The radioconjugates were purified using size exclusion NAP-5 columns providing radiochemical purities over 98%. Both [^68^Ga]Ga-Ec1-NODAGA and [^68^Ga]Ga-Ec1-NOTA demonstrated high stability during incubation with a large excess of EDTA for up to 3 h, while [^68^Ga]Ga-Ec1-DOTA released around 25% of activity after 1 h of incubation in EDTA (Table 2).

**Table 1:** Radiolabeling of Ec1 variants with ^68^Ga

	Radiochemical yield (RCY), %	RCY, after EDTA incubation	Radiochemical purity (RCP), %
Ec1-DOTA (n = 5)	39 ± 14	38 ± 14	100 ± 0
Ec1-NODAGA (n = 4)	88 ± 12	90 ± 8	100 ± 0
Ec1-NOTA (n = 5)	85 ± 7	83 ± 9	100 ± 0

**Table 2:** In vitro stability of ^68^Ga-labelled EpCAM Ec1 variants

	Protein-associated activity, %			
	1 h		3 h	
	PBS	EDTA	PBS	EDTA
Ec1-DOTA	94 ± 2	76 ± 1	93 ± 1	72 ± 2
Ec1-NODAGA	97 ± 1	94 ± 1	95.6 ± 0.2	93 ± 2
Ec1-NOTA	96.8 ± 0.2	95 ± 1	97 ± 1	94 ± 1

The stability test was performed in triplicates

^68^Ga[Ga]-Ec1-DOTA had the lowest stability in mouse serum both at 1 h and 3 h of incubation compared to ^68^Ga[Ga]-Ec1-NOTA and ^68^Ga[Ga]-Ec1-NODAGA (Table 3). After separation of samples on NAP-5, more activity (ca. 4–6%) was collected in the high-molecular weight fraction for serum samples compared to PBS samples. This indicates that a fraction of released gallium-68 could transchelate to serum proteins after 3 h of incubation. For the control samples containing ^68^Ga[Ga]Cl_3_ in serum, ca. 70% of activity was transchelated to serum proteins after 3 h of incubation.

**Table 3:** Serum stability of ^68^Ga[Ga]-labelled Ec1 conjugates analyzed by iTLC after 1 and 3 h of incubation at 37 °C

	Protein-associated activity, %					
	1 h (iTLC)		3 h (iTLC)		3 h (NAP-5)	
	PBS	Mouse serum	PBS	Mouse serum	PBS	Mouse serum
^68^Ga[Ga]-Ec1-DOTA	90 ± 0	92 ± 0	91 ± 1	90 ± 2	85 ± 0	92 ± 1
^68^Ga[Ga]-Ec1-NODAGA	93 ± 0	97 ± 0	91 ± 1	96 ± 0	89 ± 0	95 ± 0
^68^Ga[Ga]-Ec1-NOTA	95 ± 0	97 ± 0	92 ± 0	96 ± 0	91 ± 0	95 ± 0
^68^Ga[Ga]Cl_3_	1 ± 0	0.4 ± 0.1	5 ± 4	2 ± 1	12 ± 0	70 ± 1

After 3 h, the samples were loaded on NAP-5 column and the fractions corresponding to high molecular weight components (protein-associated activity) and low molecular weight components were collected. Control samples were incubated in PBS, ^68^Ga[Ga]Cl_3_ was included for reference. The data is presented as an average (n = 2) ± SD

**Fig. 2:**
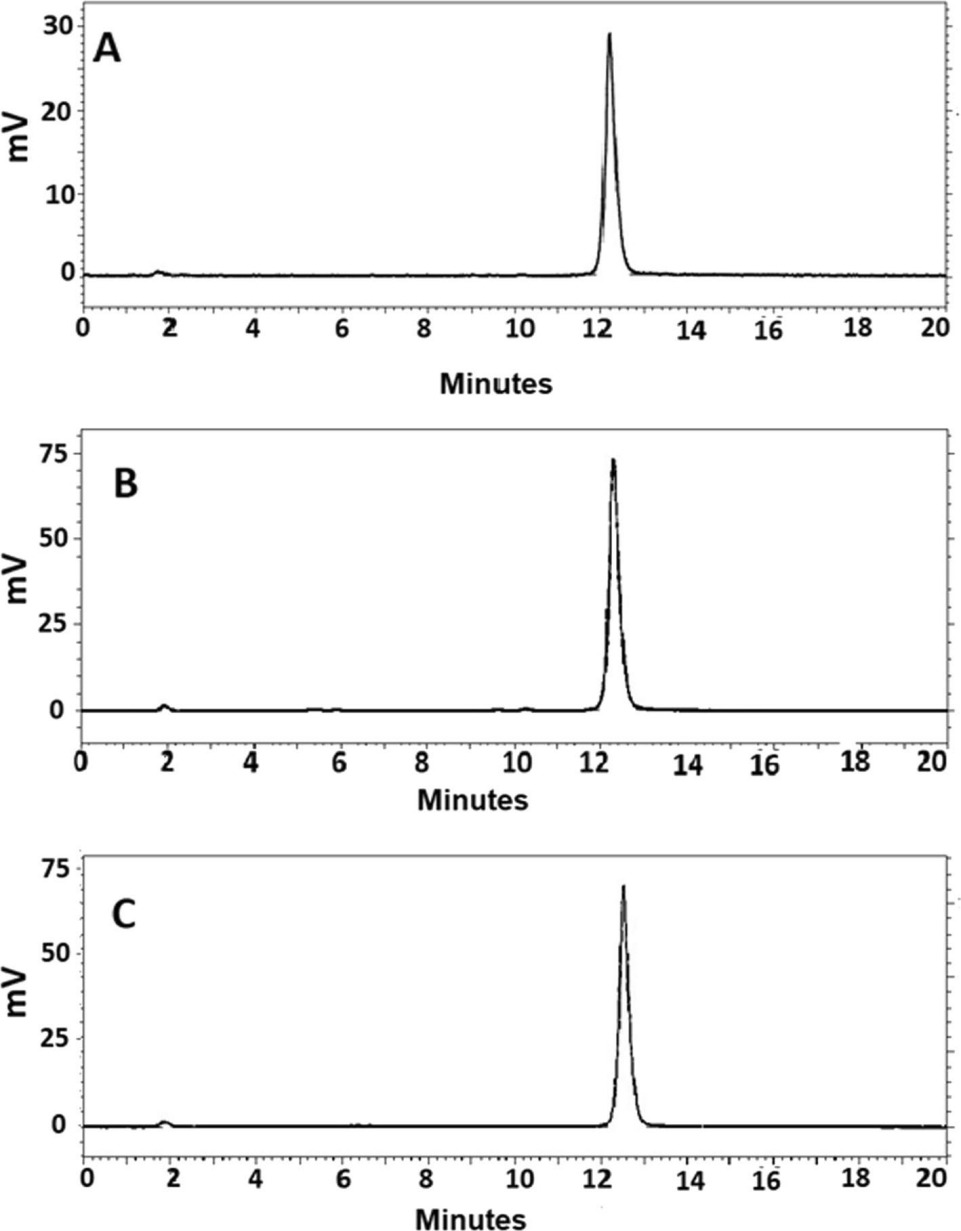
Reversed-phase radio-HPLC chromatograms of **A** [^68^Ga]Ga-Ec1-DOTA, **B** [^68^Ga]Ga-Ec1-NODAGA and **C** [^68^Ga]Ga-Ec1-NOTA

According to the radio-HPLC radiochromatograms (Fig. 2), the retention time of the radiolabelled conjugates was around 12 min. Only one major peak was observed for each radiolabelled compound.

### In vitro studies

**Fig. 3:**
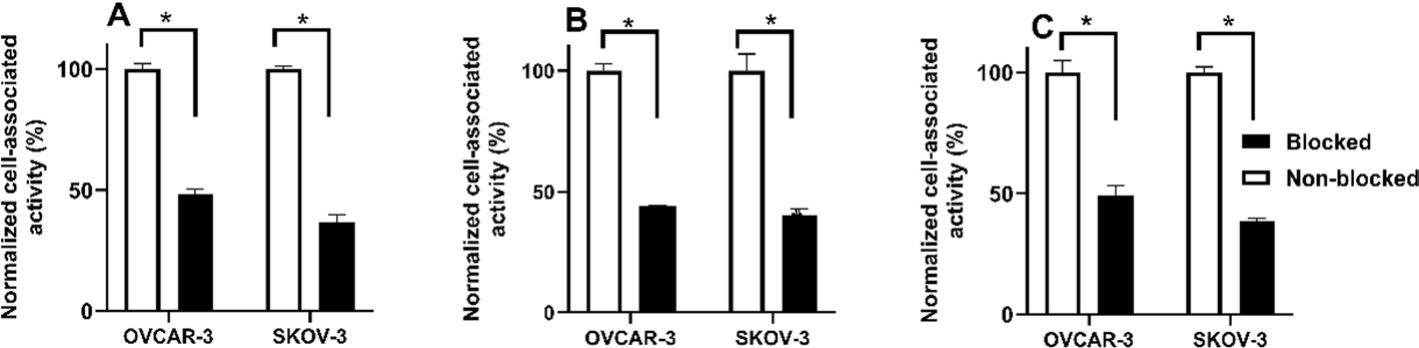
In vitro binding specificity of **A** [^68^Ga]Ga-Ec1-DOTA, **B** [^68^Ga]Ga-Ec1-NODAGA and **C** [^68^Ga]Ga-Ec1-NOTA to EpCAM-expressing SKOV-3 and OVCAR-3 cells. The data are presented as an average value (n = 3) ± standard deviation (SD). Asterisks mark (*) shows a significant difference between values (*p* < 0.05)

An in vitro binding specificity assay was performed using EpCAM-expressing SKOV-3 and OVCAR-3 ovarian cancer cells. Blocking the EpCAM receptors with an excess of non-labelled Ec1 resulted in a significant (*p* < 0.05) decrease of binding of all ^68^Ga-labelled Ec1 conjugates in the blocked groups compared to the nonblocked ones (Fig. 3). This demonstrated that the binding was EpCAM-mediated in vitro.

**Fig. 4:**
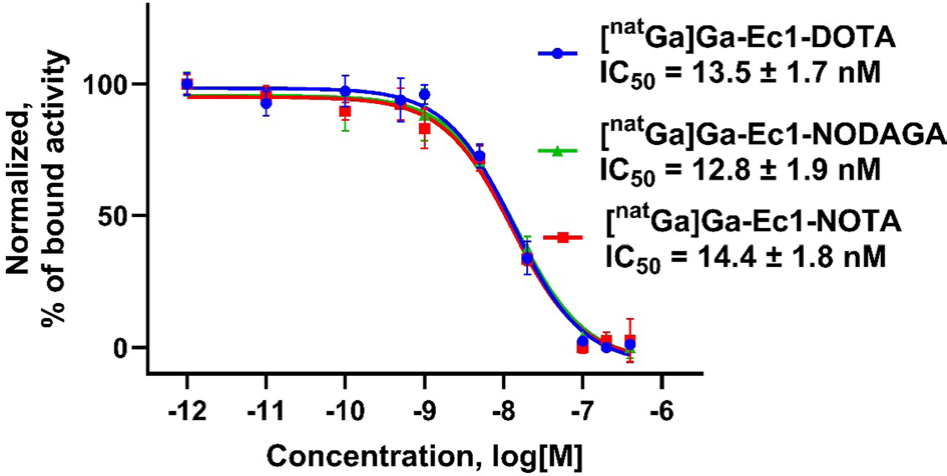
Inhibition of [^111^In]In-Ec1-DOTA binding to SKOV-3 cells by [^nat^Ga]Ga-Ec1-X [X = DOTA (●), NODAGA (▲), and NOTA (■)]. Data are presented as mean value (n = 3) ± standard deviation and normalized to the highest value for Ec1-DOTA

To compare the relative binding strength of Ec1-X (X = DOTA, NOTA and NODAGA), they were loaded with natural gallium and IC_50_ was measured using competitive binding with [^111^In]In-Ec1-DOTA in SKOV-3 cells (Fig. 4). The IC_50_ values were in nanomolar range.. There were no statistically significant differences between the IC_50_ values (*p* > 0.05 for all comparisons).

**Fig. 5:**
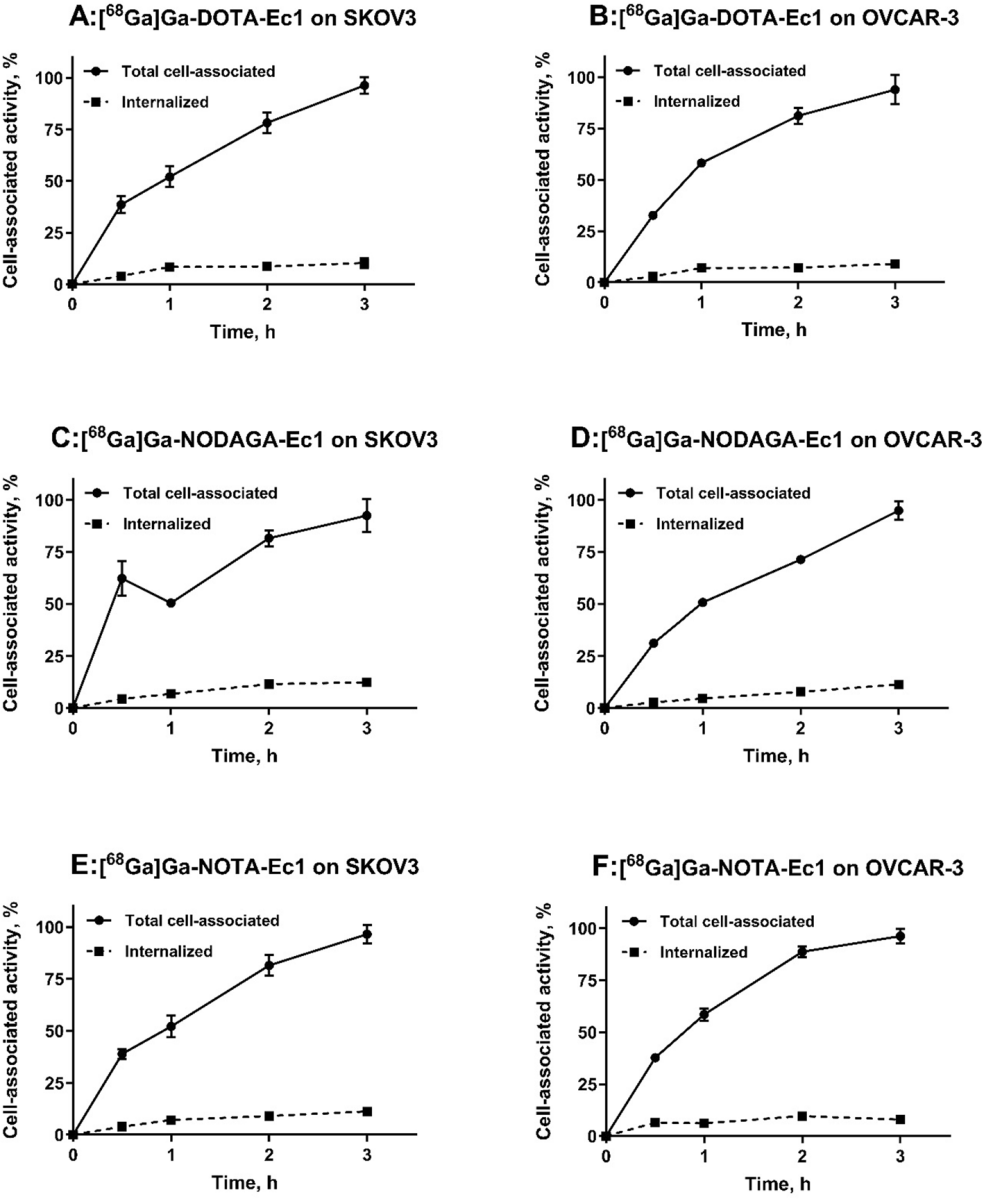
Cellular processing of [^68^Ga]Ga-Ec1-DOTA (**A**, **B**), [^68^Ga]Ga-Ec1-NODAGA (**C**, **D**) and [^68^Ga]Ga-Ec1-NOTA (**E**, **F**) in EpCAM-expressing cells. The data are presented as an average (n = 3) and SD. Error bars might not be visible when they are smaller than symbols

The cellular processing data (Fig. 5) showed that the pattern of binding and internalization was similar for all radioconjugates. The cell-associated activity increased during the incubation. The internalization was relatively slow, ranging from 8 to 12% of the total cell-associated activity after 3 h incubation.

### In vivo studies

**Fig. 6:**
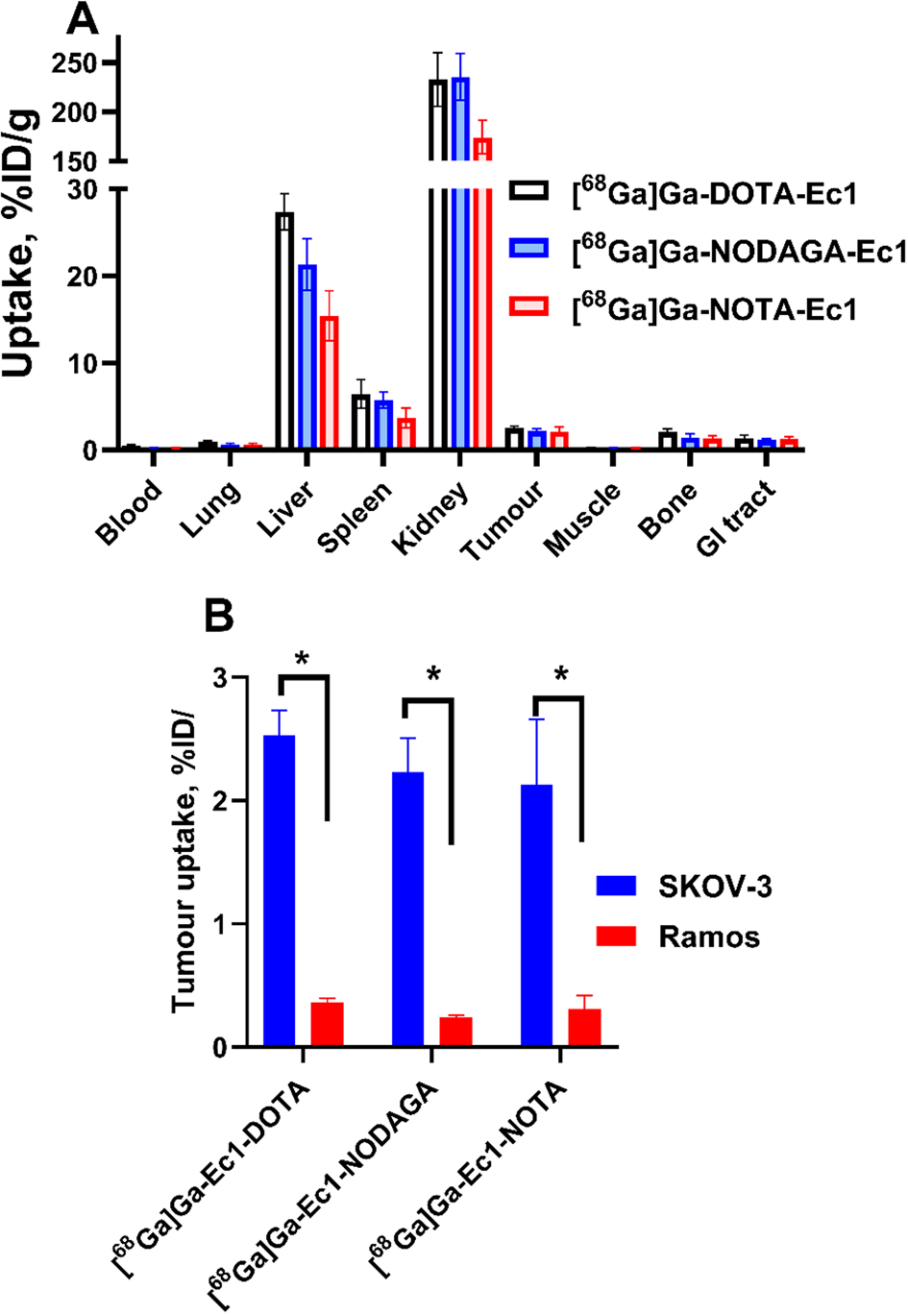
**A** Comparative biodistribution of ^68^Ga-labelled Ec1 conjugates in BALB/c nu/nu mice bearing SKOV-3 xenografts 3 h after injection. **B** Uptake of [^68^Ga]Ga-Ec1-DOTA, [^68^Ga]Ga-Ec1-NODAGA and [^68^Ga]Ga-Ec1-NOTA in SKOV-3 (EpCAM-positive) and Ramos (EpCAM-negative) xenografts 3 h after injection. Data are expressed as %ID/g and are an average from four mice (n = 4) ± SD. Asterisks mark (*) shows a significant difference between values (*p* < 0.05). Data for [^68^Ga]Ga-Ec1-DOTA in Ramos xenografts were taken from Deyev et al. (2021)

Biodistribution of [^68^Ga]Ga-Ec1-DOTA, [^68^Ga]Ga-Ec1-NOTA and [^68^Ga]Ga-Ec1-NODAGA in BALB/c nu/nu mice bearing human cancer xenografts demonstrated that the uptake of all ^68^Ga-labelled conjugates was significantly (*p* < 0.05) higher in EpCAM-positive SKOV-3 xenografts than in EpCAM-negative Ramos xenografts 3 h after injection (Fig. 6), demonstrating in vivo EpCAM-specific accumulation in tumors.

The full biodistribution data is shown in Table 4. [^68^Ga]Ga-Ec1-NOTA showed significantly (*p* < 0.05) lower blood concentration than [^68^Ga]Ga-Ec1-DOTA (0.21 ± 0.05 and 0.52 ± 0.10%ID/g, respectively). The uptake in most normal organs and tissues followed the same trend as the blood concentration. There was a tendency of lower uptake in almost all organs and tissues for [^68^Ga]Ga-Ec1-NOTA. The uptake in liver was significantly (*p* < 0.05) lower for [^68^Ga]Ga-Ec1-NOTA (16 ± 3%ID/g) than for [^68^Ga]Ga-Ec1-DOTA (27 ± 2%ID/g) and [^68^Ga]Ga-Ec1-NODAGA (21 ± 3%ID/g). The uptake of [^68^Ga]Ga-Ec1-NODAGA was also significantly (*p* < 0.05) lower in blood and liver compared with [^68^Ga]Ga-Ec1-DOTA, with no significant differences detected in other normal organs and tissues. No significant differences in tumor uptake were observed among the conjugates. However, a significantly (*p* < 0.05) higher tumor-to-blood ratio for [^68^Ga]Ga-Ec1-NOTA (10 ± 3) than for [^68^Ga]Ga-Ec1-DOTA (5 ± 1) was observed. There was a tendency of higher tumor-to-organ ratios for [^68^Ga]Ga-Ec1-NOTA than for the other two radioconjugates (Table 5).

**Table 4:** Comparative biodistribution of ^68^Ga-labelled Ec1 conjugates in BALB/c nu/nu mice bearing SKOV-3 and Ramos xenografts 3 h after injection

	[^68^Ga]Ga-Ec1-DOTA	[^68^Ga]Ga-Ec1-NODAGA	[^68^Ga]Ga-Ec1-NOTA
Blood	0.52 ± 0.10^a,b^	0.26 ± 0.04^a^	0.21 ± 0.05^b^
Lung	0.9 ± 0.2	0.6 ± 0.2	0.6 ± 0.2
Liver	27 ± 2^a,b^	21 ± 3^a,c^	16 ± 3^b,c^
Spleen	6.4 ± 1.7^b^	5.8 ± 1.0	3.7 ± 1.1^b^
Kidney	233 ± 27^b^	236 ± 24^c^	174 ± 17^b,c^
Tumor (SKOV3, EpCAM+)	2.5 ± 0.2	2.2 ± 0.3	2.1 ± 0.5
Tumor (Ramos, EpCAM−)	0.36 ± 0.04	0.24 ± 0.02	0.3 ± 0.1
Muscle	0.28 ± 0.06	0.24 ± 0.02	0.22 ± 0.03
Bone	2.1 ± 0.4^b^	1.5 ± 0.4	1.4 ± 0.3^b^
GI tract**	1.4 ± 0.3	1.2 ± 0.2	1.2 ± 0.3
Carcass**	9 ± 1	8 ± 1	9 ± 2

Data are expressed as the percentage of injected dose per gram of tissue (% ID/g). The data are presented as the average value (n = 3–4) ± SD

**Data for gastrointestinal (GI) tract with content and carcass are presented as % of injected dose per whole sample

^a^Significant difference (*p* < 0.05) between [^68^Ga]Ga-Ec1-DOTA and [^68^Ga]Ga-Ec1-NODAGA

^b^Significant difference (*p* < 0.05) between [^68^Ga]Ga-Ec1-DOTA and [^68^Ga]Ga-Ec1-NOTA

^c^Significant difference (*p* < 0.05) between [^68^Ga]Ga-Ec1-NODAGA and [^68^Ga]Ga-Ec1-NOTA. One-way ANOVA with Bonferroni's multiple comparisons test was performed to determine significant (*p* < 0.05) differences

**Table 5:** Tumor-to-organ ratios of ^68^Ga-labelled Ec1 conjugates in BALB/c nu/nu mice bearing SKOV-3 xenografts 3 h after injection

	[^68^Ga]Ga-Ec1-DOTA	[^68^Ga]Ga-Ec1-NODAGA	[^68^Ga]Ga-Ec1-NOTA
Blood	5 ± 1^a^	9 ± 2	10 ± 3^a^
Lung	2.8 ± 0.6	3.6 ± 0.5	3.8 ± 1.8
Liver	0.093 ± 0.004	0.105 ± 0.002	0.144 ± 0.055
Spleen	0.045 ± 0.009	0.041 ± 0.004	0.064 ± 0.027
Kidney	0.011 ± 0.002	0.010 ± 0.002	0.012 ± 0.004
Muscle	9.3 ± 1.5	9.5 ± 1.5	10.0 ± 2.6
Bone	1.3 ± 0.3	1.6 ± 0.5	1.6 ± 0.6

The data are presented as the average (n = 3–4) and SD

^a^Significant difference (*p* < 0.05) between **[**^68^Ga]Ga-Ec1-DOTA and [^68^Ga]Ga-Ec1-NOTA. One-way ANOVA with Bonferroni's multiple comparisons test was performed to determine significant (*p* < 0.05) differences

**Fig. 7:**
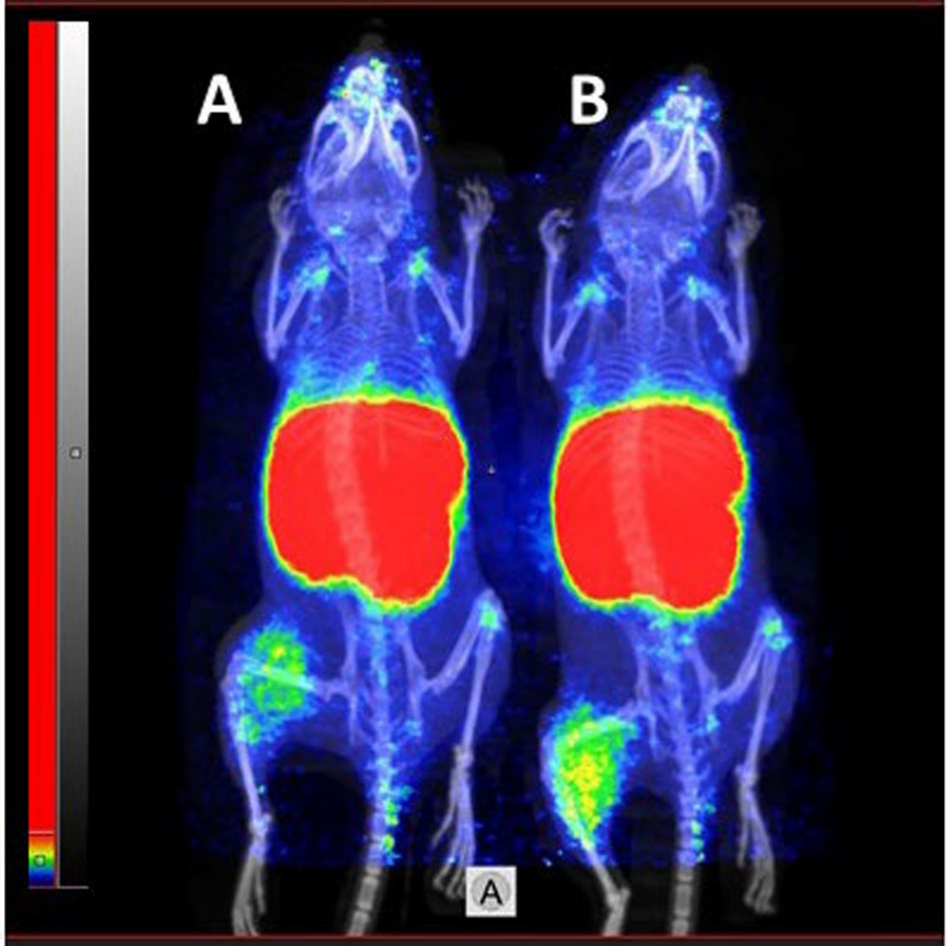
NanoPET/CT imaging of EpCAM expression using **A** [^68^Ga]Ga-Ec1-DOTA and **B** [^68^Ga]Ga-Ec1-NOTA in BALB/c nu/nu mice bearing SKOV-3 xenografts 3 h after injection

Results of nanoPET/CT imaging (Fig. 7) of [^68^Ga]Ga-Ec1-DOTA and [^68^Ga]Ga-Ec1-NOTA in BALB/c nu/nu mice bearing EpCAM-positive SKOV-3 xenografts 3 h after injection confirmed the ex vivo biodistribution data. Clear visualization of EpCAM-expressing xenografts was observed using the both tracers.

## Discussion

Elevated expression of EpCAM can drive sustained proliferation and tumor or metastatic growth, potentially leading to poor prognosis for the patient (Ko et al. 2018). Its link to clinical outcomes is complex and may vary based on the tumor's origin or the stage of tumor progression (Herreros-Pomares et al. 2018). Targeted drug delivery to cancer-associated cell surface proteins might enhance treatment selectivity. Radionuclide-based imaging of molecular therapeutic targets can aid in stratifying patients for the targeted treatments (Hofman et al. 2020; Mileva et al. 2024). Several studies have shown that the molecular design e.g. differences in structure of the metal/chelator-complex, surface exposure of functional groups and local distribution of charge, could affect the biodistribution and targeting properties of small targeting agents used for imaging of different molecular targets (Tolmachev and Orlova 2010; Strand et al. 2013; Lindbo et al. 2018; Tolmachev et al. 2018; von Witting et al. 2019; Mitran et al. 2019; Rinne et al. 2019a, b; Leitao et al. 2019; Deyev et al. 2021). In a previous study we have identified that attachment of DOTA at the C-terminus of Ec1 is preferrable to the N-terminal for attachment, providing lower uptake in normal organs (Deyev et al. 2021). However, the performance of [^68^Ga]Ga-Ec1-DOTA was suboptimal compared to the other studied radiometals, indium-111 and cobalt-57. Thus, in this study, we aimed to investigate the influence of radiometal-chelate complex on biodistribution and targeting properties of Ec1 labelled with ^68^Ga for preclinical imaging of EpCAM-expressing tumors using PET. Three different macrocyclic chelators, DOTA, NOTA and NODAGA chelators, were site-specifically conjugated to the C-terminus of Ec1 for labeling with ^68^Ga. The short half-life of ^68^Ga permits imaging a few hours after injection, which fits well with DARPins, since they have fast clearance from blood and good accumulation in tumors at early time points. As observed in the previous study and also in this study, labeling of Ec1-DOTA with gallium-68 was performed with low radiochemical yield, and a weaker radiolabel stability was noted for this radioconjugate. The use of NOTA and NODAGA provided a major improvement both in the radiochemical yields and label stabilities (Tables 1, 2, 3). A lower radiochemical yield for [^68^Ga]Ga-Ec1-DOTA than for [^68^Ga]Ga-Ec1-NOTA and [^68^Ga]Ga-Ec1-NODAGA can be attributed to several factors, including chelator ring size, chelation kinetics, thermodynamic stability, and labeling conditions. DOTA has a 12-membered ring and its large ring size does not optimally match the coordination preferences of Ga^3+^, potentially leading to less efficient complexation (Wadas et al. 2010). DOTA requires elevated temperatures (typically 80–100 °C) and longer reaction times for efficient labeling. The slower kinetics can lead to lower radiochemical yields under suboptimal conditions. NOTA and NODAGA have smaller ring sizes (9-membered) and are better suited for the ionic radius of Ga^3+^. Studies comparing the labeling efficiencies of DOTA, NOTA, and NODAGA with gallium-68 demonstrated that NOTA and NODAGA provide higher radiochemical yields and specific activities under similar conditions (Notni et al. 2012; Tsionou et al. 2017). A lower radiochemical yield for [^68^Ga]Ga-Ec1-DOTA in our study can also be due to the relatively low temperature of radiolabeling limited to 60 °C. Further increasing the labeling temperature was not possible as it results in a significant loss of Ec1 binding to EpCAM-expressing cells (Deyev et al. 2021).

Binding of ^68^Ga-labelled Ec1 conjugates was target-mediated in vitro (Fig. 3). The binding properties of Ec1 variants were evaluated in a competitive binding assay showing IC_50_ values in the low nanomolar range for all conjugates (Fig. 4) without any significant differences. This result suggests that there is no substantial influence of the chelator on the binding affinity. The cellular processing study of [^68^Ga]Ga-Ec1-DOTA, [^68^Ga]Ga-Ec1-NODAGA and [^68^Ga]Ga-Ec1-NOTA (Fig. 5) showed slow internalization and similar processing patterns for all three conjugates, suggesting that there is no considerable impact of the chelator on the cellular processing of the conjugates. All the conjugates targeted specifically EpCAM-expressing xenografts in mice, as the tumor uptake was significantly lower in Ramos xenografts (EpCAM-negative) compared with SKOV-3 xenografts (EpCAM-positive).

Biodistribution data in tumor-bearing mice 3 h after injection (Table 4) demonstrated that the use of [^68^Ga]Ga-Ec1-NOTA provided lower uptake in most normal organs in vivo without compromising the tumor uptake. Importantly, the blood concentration of [^68^Ga]Ga-Ec1-NOTA was 2.5-fold lower than for the previously reported [^68^Ga]Ga-Ec1-DOTA. The delayed blood clearance of [^68^Ga]Ga-DOTA-Ec1 could be due to its low stability. A release of activity under EDTA challenge and when incubated with mouse serum was observed for this conjugate in vitro. A part of the released gallium-68 could bind to blood proteins and accumulate in bone resulting in delayed blood clearance and elevated bone uptake. Even minor changes in the labeling strategy of ESPs have previously been shown to impact their biodistribution profile (Tolmachev and Orlova 2020). Choosing an optimal labeling strategy could therefore improve the biodistribution (Knetsch et al. 2013; Li Y et al. 2022b;). The biodistribution results of [^68^Ga]Ga-Ec1-NOTA showed a noticeable reduction in activity uptake in liver and spleen (1.7-fold), bone (1.5-fold), as well as kidneys (1.4-fold) in comparison to [^68^Ga]Ga-Ec1-DOTA 3 h after injection. 

We previously investigated whether the renal uptake of DARPins radiolabeled with technetium-99 m tricarbonyl can be reduced using co-administration of compounds that act on various parts of the reabsorption system in the kidney. We found that common clinical strategies, such as co-injection of lysine or gelofusine, were not effective for the reduction of their kidney uptake and that the uptake was regulated by an ATP-driven mechanism independent of DARPin structure and binding site composition (Altai et al. 2020). As the exact mechanism of renal uptake of DARPins is not known, it can be suggested that local charge and spatial geometry of the radiometal-chelator complex may influence interaction with scavenger receptors, renal absorption and retention of radiocatabolites in the kidneys.

We previously observed that the position of DOTA chelator (N- or C-termini) in [^68^Ga]Ga-Ec1-DOTA had an influence on its uptake in normal organs, with N-terminal placement providing lower kidney uptake at 3 h p.i. (175 ± 9%ID/g vs. 198 ± 8%ID/g for N- and C-terminal placement) (Deyev et al. 2021). In this study, we have investigated the influence of a chelator type placed at the C terminus of Ec1. The overall charge of NOTA, NODAGA and DOTA complexes with gallium-68 is + 1, 0, and 0, respectively. The coordination geometry of complexes is also different for all chelators. Both NOTA and NODAGA are triaza-chelators. NODAGA is a derivative of NOTA with an additional carboxylic arm that brings the overall complex charge from + 1 to 0. It has been observed for HER3-targeting affibody molecule (HE)_3_-Z_08698_ that positively charged [^68^Ga]Ga-NOTA in [^68^Ga]Ga-(HE)_3_-Z_08698_-NOTA reduced its kidney uptake to 138 ± 28%ID/g in comparison to the neutrally charged [^68^Ga]Ga-NODAGA in [^68^Ga]Ga-(HE)_3_-Z_08698_-NODAGA having 324 ± 11%ID/g kidney uptake at 3 h p.i. (Dahlsson Leitao et al. 2019). In another study, [^68^Ga]Ga-NOTA-PEG2-RM26 peptide also had significantly lower radioactivity accumulation in the kidneys (1.9 ± 0.3%ID/g) in comparison with NODAGA (2.7 ± 0.3%ID/g) and DOTAGA (2.9 ± 0.3%ID/g) derivatives at 2 h p.i. (Varasteh et al. 2015). 

Liver was the second highest organ of uptake after kidneys for all Ec1 conjugates, with 27 ± 2%ID/g for ^68^Ga[Ga]-Ec1-DOTA, 21 ± 3%ID/g for ^68^Ga[Ga]-Ec1-NODAGA and 16 ± 3%ID/g for ^68^Ga[Ga]-Ec1-NOTA. In the adult mouse liver, mature hepatocytes typically do not express EpCAM (Tanaka et al. 2009). It is more likely that the liver uptake is mediated by non-specific interactions. The differences in uptake and retention of activity might be due to the differences in local charge and spatial geometry of the radiometal-chelator complexes. In our previous study, C-terminal position of ^68^Ga[Ga]-DOTA provided lower uptake of Ec1 in liver than N-terminal position (Deyev et al. 2021). Compared to the other radiometals, [^68^Ga]Ga-labeled variants had the highest uptake in liver, followed by the variants labeled with cobalt-57 and indium-111. Another potential reason for higher liver uptake of ^68^Ga[Ga]-Ec1-DOTA could be its lower stability compared to the other radioconjugates. The colloidal gallium content was between 0.1–0.3% for all radioconjugates.

The tumor-to-blood ratio is an essential parameter since the activity in blood reduces the imaging contrast. In this study, [^68^Ga]Ga-Ec1-NOTA provided the tumor-to-blood ratio of 10 ± 3 only 3 h after injection, which is approximately twofold higher than for the previously reported DOTA analogue, which potentially leads to lower background signal and improved imaging contrast at early time point after injection.

## Conclusion

Optimizing the labeling strategy, such as selecting the optimal radiometal-chelate complex, could help reduce off-target interactions and improve the imaging contrast of Ec1 agents. In this study, [^68^Ga]Ga-Ec1-NOTA demonstrated the highest tumor-to-blood ratio, suggesting a potential advantage for visualizing EpCAM-expressing tumors. Additionally, [^68^Ga]Ga-Ec1-NOTA had the lowest uptake in normal organs, such as the liver, which could be beneficial for imaging applications. These findings indicate that [^68^Ga]Ga-Ec1-NOTA is a promising candidate for imaging of EpCAM expression using Ec1.

## Data Availability

Data will be made available on reasonable request.

## References

[CR1] Alhuseinalkhudhur A, Lindman H, Liss P, Sundin T, Frejd FY, Hartman J, Iyer V, Feldwisch J, Lubberink M, Rönnlund C, Tolmachev V, Velikyan I, Sörensen J. Human epidermal growth factor receptor 2-targeting [^68^Ga]Ga-ABY-025 PET/CT predicts early metabolic response in metastatic breast cancer. J Nucl Med. 2023;64:1364–70. 37442602 10.2967/jnumed.122.265364 PMC10478820

[CR2] Altai M, Garousi J, Rinne SS, Schulga A, Deyev S, Vorobyeva A. On the prevention of kidney uptake of radiolabeled DARPins. EJNMMI Res. 2020;4(10):7. 10.1186/s13550-020-0599-1 PMC7000568 32020413

[CR3] Altena R, Burén SA, Blomgren A, Karlsson E, Tzortzakakis A, Brun N, Moein MM, Jussing E, Frejd FY, Bergh J, Tran TA, Hartman J, Axelsson R. Human epidermal growth factor receptor 2 (HER2) PET imaging of HER2-low breast cancer with [68Ga]Ga-ABY-025: results from a pilot study. J Nucl Med. 2024;65:700–7. 38548353 10.2967/jnumed.123.266847

[CR5] Altena R, Burén SA, Tran T, Axelsson R. HER2-low breast cancer can be visualized by HER2 PET. J Nucl Med. 2023;64:1841. 37709535 10.2967/jnumed.123.266101

[CR4] Asti M, Iori M, Capponi PC, Atti G, Rubagotti S, Martin R, et al. Influence of different chelators on the radiochemical properties of a 68-gallium labelled Bombesin analogue. Nucl Med Biol. 2014;41:24–35. 24183610 10.1016/j.nucmedbio.2013.08.010

[CR6] Binz HK, Stumpp MT, Forrer P, Amstutz P, Plückthun A. Designing repeat proteins: well-expressed, soluble and stable proteins from combinatorial libraries of consensus Ankyrin repeat proteins. J Mol Biol. 2003;332:489–503. 12948497 10.1016/s0022-2836(03)00896-9

[CR7] Binz HK, Amstutz P, Kohl A, Stumpp MT, Briand C, Forrer P, Grütter MG, Plückthun A. High-affinity binders selected from designed Ankyrin repeat protein libraries. Nat Biotechnol. 2004;22:575–82. 15097997 10.1038/nbt962

[CR8] Binz HK, Bakker TR, Phillips DJ, Cornelius A, Zitt C, Göttler T, Sigrist G, Fiedler U, Ekawardhani S, Dolado I, Saliba JA, Tresch G, Proba K, Stumpp MT. Design and characterization of MP0250, a tri-specific anti-HGF/anti-VEGF DARPin® drug candidate. Mabs. 2017;8:1262–9. 10.1080/19420862.2017.1305529 PMC5680794 29035637

[CR9] Bragina O, von Witting E, Garousi J, Zelchan R, Sandström M, Orlova A, Medvedeva A, Doroshenko A, Vorobyeva A, Lindbo S, Borin J, Tarabanovskaya N, Sörensen J, Hober S, Chernov V, Tolmachev V. Phase I study of ^99m^Tc-ADAPT6, a scaffold protein-based probe for visualization of HER2 expression in breast cancer. J Nucl Med. 2021;62:493–9. 32817142 10.2967/jnumed.120.248799 PMC8049361

[CR10] Bragina O, Chernov V, Schulga A, Konovalova E, Garbukov E, Vorobyeva A, Orlova A, Tashireva L, Sörensen J, Zelchan R, Medvedeva A, Deyev S, Tolmachev V. Phase I trial of ^99m^Tc-(HE)3–G3, a DARPin-based probe for imaging of HER2 expression in breast cancer. J Nucl Med. 2022;63:528–35. 34385343 10.2967/jnumed.121.262542 PMC8973295

[CR11] Breitz HB, Tyler A, Bjorn MJ, Lesley T, Weiden PL. Clinical experience with Tc-99m nofetumomab merpentan (Verluma) radioimmunoscintigraphy. Clin Nucl Med. 1997;22:615–20. 9298295 10.1097/00003072-199709000-00007

[CR12] Cui Y, Li J, Liu X, Gu L, Lyu M, Zhou J, Zhang X, Liu Y, Zhu H, Zhang T, Sun F. Dynamic expression of EpCAM in primary and metastatic lung cancer is controlled by both genetic and epigenetic mechanisms. Cancers (Basel). 2022;14:4121. 36077658 10.3390/cancers14174121 PMC9454530

[CR13] Dahlsson Leitao C, Rinne SS, Mitran B, Vorobyeva A, Andersson KG, Tolmachev V, Ståhl S, Löfblom J, Orlova A. Molecular design of HER3-targeting affibody molecules: influence of chelator and presence of HEHEHE-tag on biodistribution of 68Ga-labeled tracers. Int J Mol Sci. 2019;20:1080. 30832342 10.3390/ijms20051080 PMC6429182

[CR14] Deyev SM, Vorobyeva A, Schulga A, Abouzayed A, Güntherd T, Garousi J, Konovalova E, Dinge H, Gräslund T, Orlova A, Tolmachev V. Effect of a radiolabel biochemical nature on tumor-targeting properties of EpCAM-binding engineered scaffold protein DARPin Ec1. Int J Biol Macromol. 2020;15:216–25. 10.1016/j.ijbiomac.2019.12.147 31863835

[CR15] Deyev SM, Xu T, Liu Y, Schulga A, Konovalova E, Garousi J, Rinne SS, Larkina M, Ding H, Gräslund T, Orlova A, Tolmachev V, Vorobyeva A. Influence of the position and composition of radiometals and radioiodine labels on imaging of Epcam expression in prostate cancer model using the DARPin Ec1. Cancers (Basel). 2021;13:3589. 34298801 10.3390/cancers13143589 PMC8304184

[CR16] Eder M, Knackmuss S, Le Gall F, Reusch U, Rybin V, Little M, Haberkorn U, Mier W, Eisenhut M. ^68^Ga-labelled recombinant antibody variants for immuno-PET imaging of solid tumors. Eur J Nucl Med Mol Imaging. 2010;37:1397–407. 20157706 10.1007/s00259-010-1392-6

[CR17] Eissler N, Altena R, Alhuseinalkhudhur A, Bragina O, Feldwisch J, Wuerth G, Loftenius A, Brun N, Axelsson R, Tolmachev V, Sörensen J, Frejd FY. Affibody PET imaging of HER2-expressing cancers as a key to guide HER2-targeted therapy. Biomedicines. 2024;12:1088. 38791050 10.3390/biomedicines12051088 PMC11118066

[CR19] Fani M, André JP, Maecke HR. ^68^Ga-PET: a powerful generator-based alternative to cyclotron-based PET radiopharmaceuticals. Contrast Media Mol Imaging. 2008;3:53–63. 18383558 10.1002/cmmi.232

[CR20] Garousi J, Orlova A, Frejd FY, Tolmachev V. Imaging using radiolabelled targeted proteins: radioimmunodetection and beyond. EJNMMI Radiopharm Chem. 2020;5:16. 32577943 10.1186/s41181-020-00094-w PMC7311618

[CR21] Gires O, Pan M, Schinke H, Canis M, Baeuerle PA. Expression and function of epithelial cell adhesion molecule EpCAM: where are we after 40 years? Cancer Metastasis Rev. 2020;39:969–87. 32507912 10.1007/s10555-020-09898-3 PMC7497325

[CR22] Hall MA, Pinkston KL, Wilganowski N, Robinson H, Ghosh P, Azhdarinia A, Vazquez-Arreguin K, Kolonin AM, Harvey BR, Sevick-Muraca EM. Comparison of mAbs targeting epithelial cell adhesion molecule for the detection of prostate cancer lymph node metastases with multimodal contrast agents: quantitative small-animal PET/CT and NIRF. J Nucl Med. 2012;53:1427–37. 22872743 10.2967/jnumed.112.106302

[CR23] Harris WR, Pecoraro VL. Thermodynamic binding constants for gallium transferrin. Biochemistry. 1983;22:292–9. 6402006 10.1021/bi00271a010

[CR24] Herreros-Pomares A, Aguilar-Gallardo C, Calabuig-Fariñas S, Sirera R, Jantus-Lewintre E, Camps C. EpCAM duality becomes this molecule in a new Dr. Jekyll and Mr. Hyde tale. Crit Rev Oncol Hematol. 2018;126:52–63. 29759567 10.1016/j.critrevonc.2018.03.006

[CR25] Hofman MS, Lawrentschuk N, Francis RJ, Tang C, Vela I, Thomas P, Rutherford N, Martin JM, Frydenberg M, Shakher R, Wong LM, Taubman K, Ting Lee S, Hsiao E, Roach P, Nottage M, Kirkwood I, Hayne D, Link E, Marusic P, Matera A, Herschtal A, Iravani A, Hicks RJ, Williams S, Murphy DG, proPSMA Study Group Collaborators. Prostate-specific membrane antigen PET-CT in patients with high-risk prostate cancer before curative-intent surgery or radiotherapy (proPSMA): a prospective, randomised, multicentre study. Lancet. 2020;395(10231):1208–16. 32209449 10.1016/S0140-6736(20)30314-7

[CR26] Houvast RD, Badr N, March T. de Muynck DAN, Sier VQ, Schomann T, Bhairosingh S, Baart VM, Peeters JAHM, van Westen GJP, Plückthun A, Burggraaf J, Kuppen PJK, Vahrmeijer AL, Sier CFM. Preclinical evaluation of EpCAM-binding designed ankyrin repeat proteins (DARPins) as targeting moieties for bimodal near-infrared fluorescence and photoacoustic imaging of cancer. Eur J Nucl Med Mol Imaging. 2024;51:2179–92. 37642704 10.1007/s00259-023-06407-w PMC11178671

[CR27] Interlandi G, Wetzel SK, Settanni G, Plückthun A, Caflisch A. Characterization and further stabilization of designed Ankyrin repeat proteins by combining molecular dynamics simulations and experiments. J Mol Biol. 2008;375:837–54. 18048057 10.1016/j.jmb.2007.09.042

[CR28] Knetsch PA, Petrik M, Rangger C, Seidel G, Pietzsch HJ, Virgolini I, Decristoforo C, Haubner R. [⁶⁸Ga]NS₃-RGD and [⁶⁸Ga] Oxo-DO3A-RGD for imaging α(v)β₃ integrin expression: synthesis, evaluation, and comparison. Nucl Med Biol. 2013;40:65–72. 23102540 10.1016/j.nucmedbio.2012.09.006

[CR29] Ko CJ, Li CJ, Wu MY, Chu PY. Overexpression of epithelial cell adhesion molecule as a predictor of poor outcome in patients with hepatocellular carcinoma. Exp Ther Med. 2018;16:4810–6. 30542436 10.3892/etm.2018.6794 PMC6257457

[CR30] Kostelnik TI, Orvig C. Radioactive main group and rare earth metals for imaging and therapy. Chem Rev. 2019;119:902–56. 30379537 10.1021/acs.chemrev.8b00294

[CR31] Kosterink JG, de Jonge MW, Smit EF, Piers DA, Kengen RA, Postmus PE, Shochat D, Groen HJ. The HT, de Leij L. Pharmacokinetics and scintigraphy of indium-111-DTPA-MOC-31 in small-cell lung carcinoma. J Nucl Med. 1995;36:2356–62. 8523132

[CR32] Krasniqi A, D’Huyvetter M, Devoogdt N, Frejd FY, Sörensen J, Orlova A, Keyaerts M, Tolmachev V. Same-day imaging using small proteins: clinical experience and translational prospects in oncology. J Nucl Med. 2018;59:885–91. 29545374 10.2967/jnumed.117.199901

[CR33] Leitao CD, Rinne SS, Mitran B, Vorobyeva A, Andersson KG, Tolmachev V, Ståhl S, Löfblom J, Orlova A. Molecular design of HER3-targeting affibody molecules: influence of chelator and presence of HEHEHE-tag on biodistribution of 68Ga-labeled tracers. Int J Mol Sci. 2019;20:1080. 30832342 10.3390/ijms20051080 PMC6429182

[CR35] Lindbo S, Garousi J, Mitran B, Vorobyeva A, Oroujeni M, Orlova A, Hober S, Tolmachev V. Optimized molecular design of ADAPT-based HER2-imaging probes labeled with ^111^In and ^68^Ga. Mol Pharm. 2018;15:2674–83. 29865791 10.1021/acs.molpharmaceut.8b00204

[CR36] Liu Y, Wang Y, Sun S, Chen Z, Xiang S, Ding Z, Huang Z, Zhang B. Understanding the versatile roles and applications of EpCAM in cancers: from bench to bedside. Exp Hematol Oncol. 2022a;11:2022. 10.1186/s40164-022-00352-4 PMC9650829 36369033

[CR37] Liu Y, Yu S, Xu T, Bodenko V, Orlova A, Oroujeni M, et al. Preclinical evaluation of a new format of 68Ga- and 111In-labeled affibody molecule ZIGF-1R:4551 for the visualization of IGF-1R expression in malignant tumors using PET and SPECT. Pharmaceutics. 2022b;14:1475. 35890370 10.3390/pharmaceutics14071475 PMC9320461

[CR38] Liu T, Wu Y, Shi L, et al. Preclinical evaluation of [^99m^Tc]Tc-labeled anti-EpCAM nanobody for EpCAM receptor expression imaging by immuno-SPECT/CT. Eur J Nucl Med Mol Imaging. 2022c;49:1810–21. 35013776 10.1007/s00259-021-05670-z

[CR39] Lub-de Hooge MN, Kosterink JG, Perik PJ, et al. Preclinical characterisation of 111In-DTPA-trastuzumab. Br J Pharmacol. 2004;143:99–106. 15289297 10.1038/sj.bjp.0705915 PMC1575276

[CR40] Luo R, Liu H, Cheng Z. Protein scaffolds: antibody alternatives for cancer diagnosis and therapy. RSC Chem Biol. 2022;3:830–47. 35866165 10.1039/d2cb00094f PMC9257619

[CR41] Maeda H, Wu J, Sawa T, Matsumura Y, Hori K. Tumor vascular permeability and the EPR effect in macromolecular therapeutics: a review. J Control Release. 2000;65:271–84. 10699287 10.1016/s0168-3659(99)00248-5

[CR42] McLarty K, Cornelissen B, Scollard DA, Done SJ, Chun K, Reilly RM. Associations between the uptake of 111In-DTPA-trastuzumab, HER2 density and response to trastuzumab (Herceptin) in athymic mice bearing subcutaneous human tumour xenografts. Eur J Nucl Med Mol Imaging. 2009;36:81–93. 18712381 10.1007/s00259-008-0923-x

[CR43] Mileva M, de Vries EGE, Guiot T, Wimana Z, Deleu AL, Schröder CP, Lefebvre Y, Paesmans M, Stroobants S, Huizing M, Aftimos P, Tol J, Van der Graaf WTA, Oyen WJG, Vugts DJ, Menke-van der Houven van Oordt CW, Brouwers AH, Piccart-Gebhart M, Flamen P, Gebhart G. Molecular imaging predicts lack of T-DM1 response in advanced HER2-positive breast cancer (final results of ZEPHIR trial). NPJ Breast Cancer. 2024;10:4. 38184611 10.1038/s41523-023-00610-6 PMC10771456

[CR44] Mitran B, Thisgaard H, Rinne S, Hygum Dam J, Azami F, Tolmachev V, Orlova A, Rosenström U. Selection of an optimal macrocyclic chelator improves the imaging of prostate cancer using cobalt-labeled GRPR antagonist RM26. Sci Rep. 2019;9:17086. 31745219 10.1038/s41598-019-52914-y PMC6863848

[CR45] Notni J, Pohle K, Wester HJ. Comparative gallium-68 labeling of TRAP-, NOTA-, and DOTA-peptides: practical consequences for the future of gallium-68-PET. EJNMMI Res. 2012;2:28. 22682112 10.1186/2191-219X-2-28 PMC3538506

[CR46] Rahmim A, Zaidi H. PET versus SPECT: strengths, limitations and challenges. Nucl Med Commun. 2008;29:193–207. 18349789 10.1097/MNM.0b013e3282f3a515

[CR47] Rinne SS, Leitao CD, Mitran B, Bass TK, Andersson KG, Tolmachev V, Ståhl S, Löfblom J, Orlova A. Optimization of HER3 expression imaging using affibody molecules: influence of chelator for labeling with indium-111. Sci Rep. 2019a;9:655. 30679757 10.1038/s41598-018-36827-w PMC6345776

[CR48] Rinne SS, Leitao CD, Gentry J, Mitran B, Abouzayed A, Tolmachev V, Ståhl S, Löfblom J, Orlova A. Increase in negative charge of 68Ga/chelator complex reduces unspecific hepatic uptake but does not improve imaging properties of HER3-targeting affibody molecules. Sci Rep. 2019b;9:17710. 31776413 10.1038/s41598-019-54149-3 PMC6881397

[CR49] Stefan N, Martin-Killias P, Wyss-Stoeckle S, Honegger A, Zangemeister-Wittke U, Plückthun A. DARPins recognizing the tumor-associated antigen EpCAM selected by phage and ribosome display and engineered for multivalency. J Mol Biol. 2011;413:826–43. 21963989 10.1016/j.jmb.2011.09.016

[CR50] Steiner D, Forrer P, Plückthun A. Efficient selection of DARPins with sub-nanomolar affinities using SRP phage display. J Mol Biol. 2008;382:1211–27. 18706916 10.1016/j.jmb.2008.07.085

[CR51] Strand J, Honarvar H, Perols A, Orlova A, Selvaraju RK, Karlström AE, Tolmachev V. Influence of macrocyclic chelators on the targeting properties of ^68^Ga-labeled synthetic affibody molecules: comparison with ^111^In-labeled counterparts. PLoS ONE. 2013;8:e70028. 23936372 10.1371/journal.pone.0070028 PMC3731330

[CR52] Tanaka M, Okabe M, Suzuki K, Kamiya Y, Tsukahara Y, Saito S, Miyajima A. Mouse hepatoblasts at distinct developmental stages are characterized by expression of EpCAM and DLK1: drastic change of EpCAM expression during liver development. Mech Dev. 2009;126(8–9):665–76. 19527784 10.1016/j.mod.2009.06.939

[CR53] Tolmachev V, Orlova A. Influence of labelling methods on biodistribution and imaging properties of radiolabelled peptides for visualisation of molecular therapeutic targets. Curr Med Chem. 2010;17:2636–55. 20491631 10.2174/092986710791859397

[CR54] Tolmachev V, Orlova A. Affibody molecules as targeting vectors for PET imaging. Cancers. 2020;12:651. 32168760 10.3390/cancers12030651 PMC7139392

[CR55] Tolmachev T, Grönroos TJ, Yim C, Garousi J, Yue Y, Grimm S, Rajander J, Perols A, Haaparanta-Solin M, Solin O, Ferdani R, Orlova A, Anderson CJ, Karlström AE. Molecular design of radiocopper-labelled Affibody molecules. Sci Rep. 2018;8:6542. 29695813 10.1038/s41598-018-24785-2 PMC5916907

[CR56] Tsionou MI, Knapp CE, Foley CA, Munteanu CR, Cakebread A, Imberti C, et al. Comparison of macrocyclic and acyclic chelators for gallium-68 radiolabelling. RSC Adv. 2017;7:49586–99. 29308192 10.1039/c7ra09076e PMC5708347

[CR57] Varasteh Z, Mitran B, Rosenström U, Velikyan I, Rosestedt M, Lindeberg G, Sörensen J, Larhed M, Tolmachev V, Orlova A. The effect of macrocyclic chelators on the targeting properties of the 68Ga-labeled gastrin releasing peptide receptor antagonist PEG2-RM26. Nucl Med Biol. 2015;42:446–54. 25684649 10.1016/j.nucmedbio.2014.12.009

[CR58] Velikyan I. Prospective of ^68^Ga-radiopharmaceutical development. Theranostics. 2014;4:47–80. 10.7150/thno.7447 PMC3881227 24396515

[CR59] von Witting E, Garousi J, Lindbo S, Vorobyeva A, Altai M, Oroujeni M, Mitran B, Orlova A, Hober S, Tolmachev V. Selection of the optimal macrocyclic chelators for labeling with ^111^In and ^68^Ga improves contrast of HER2 imaging using engineered scaffold protein ADAPT6. Eur J Pharm Biopharm. 2019;140:109–20. 31082509 10.1016/j.ejpb.2019.05.008

[CR60] Vorobyeva A, Konovalova E, Xu T, Schulga A, Altai M, Garousi J, Rinne SS, Orlova A, Tolmachev V, Deyev S. Feasibility of imaging EpCAM expression in ovarian cancer using radiolabeled DARPin Ec1. Int J Mol Sci. 2020a;21:3310. 32392820 10.3390/ijms21093310 PMC7246691

[CR61] Vorobyeva A, Bezverkhniaia E, Konovalova E, Schulga A, Garousi J, Vorontsova O, Abouzayed A, Orlova A, Deyev SD, Tolmachev V. Radionuclide molecular imaging of EpCAM expression in triple-negative breast cancer using the Scaffold protein DARPin Ec1. Molecules. 2020b;25:4719. 33066684 10.3390/molecules25204719 PMC7587533

[CR62] Wadas TJ, Wong EH, Weisman GR, Anderson CJ. Coordinating radiometals of copper, gallium, indium, yttrium, and zirconium for PET and SPECT imaging of disease. Chem Rev. 2010;110:2858–902. 20415480 10.1021/cr900325h PMC2874951

[CR63] Wållberg H, Orlova A. Slow internalization of anti-HER2 synthetic affibody monomer 111In-DOTA-ZHER2:342-pep2: Implications for development of labelled tracers. Cancer Biother Radiopharm. 2008;23:435–42. 18771347 10.1089/cbr.2008.0464

[CR64] Warnders FJ, Waaijer SJH, Pool M, Lub-de Hooge MN, Friedrich M, Terwisscha van Scheltinga AGT, Stienen PDSK, Pieslor PC, Cheung HK, Kosterink JGW, de Vries EGE. Biodistribution and PET imaging of labeled bispecific T cell-engaging antibody targeting EpCAM. J Nucl Med. 2016;57:812–7. 26848172 10.2967/jnumed.115.168153

[CR65] Went P, Vasei M, Bubendorf L, Terracciano L, Tornillo L, Riede U, Kononen J, Simon R, Sauter G, Baeuerle PA. Frequent high-level expression of the immunotherapeutic target Ep-CAM in colon, stomach, prostate and lung cancers. Br J Cancer. 2006;94:128–35. 16404366 10.1038/sj.bjc.6602924 PMC2361083

[CR66] Wijngaarden JE, Jauw YWS, Zwezerijnen GJC, de Wit-van der Veen BJ, Vugts DJ, Zijlstra JM, van Dongen GAMS, Boellaard R, Menke-van der Houven van Oordt CW, Huisman MC. Non-specific irreversible 89Zr-mAb uptake in tumours: evidence from biopsy-proven target-negative tumours using 89Zr-immuno-PET. EJNMMI Res. 2024;14:18. 38358425 10.1186/s13550-024-01079-5 PMC10869322

[CR67] Wu AM. Engineered antibodies for molecular imaging of cancer. Methods. 2014;65:139–47. 24091005 10.1016/j.ymeth.2013.09.015 PMC3947235

[CR68] Xia L, Wu Y, Ren Y, Wang Z, Zhou N, Zhou W, Zhou L, Jia L, He C, Meng X, Zhu H, Yang Z. A whole-body imaging technique for tumor-specific diagnostics and screening of B7H3-targeted therapies. J Clin Invest. 2025;135(6): e186388. 39847434 10.1172/JCI186388 PMC11910224

[CR69] Xu D, Zhang Y, Huang W, Pan X, An S, Wang C, Huang G, Liu J, Wei W. ImmunoPET imaging of EpCAM in solid tumors with nanobody tracers: a preclinical study. Eur J Nucl Med Mol Imaging. 2025;52:388–400. 39249490 10.1007/s00259-024-06910-8

[CR70] Zahnd C, Pecorari F, Straumann N, Wyler E, Plückthun A. Selection and characterization of Her2 binding-designed Ankyrin repeat proteins. J Biol Chem. 2006;281:35167–75. 16963452 10.1074/jbc.M602547200

[CR71] Zelchan R, Chernov V, Medvedeva A, Rybina A, Bragina O, Mishina E, Larkina M, Varvashenya R, Fominykh A, Schulga A, Konovalova E, Vorobyeva A, Orlova A, Tashireva L, Deyev SM, Tolmachev V. Phase I clinical evaluation of designed ankyrin repeat protein [99mTc]Tc(CO)3-(HE)3-Ec1 for visualization of EpCAM-expressing lung cancer. Cancers. 2024;16:2815. 39199590 10.3390/cancers16162815 PMC11353007

